# *Mycobacterium tuberculosis*: Manipulator of Protective Immunity

**DOI:** 10.3390/ijms17030131

**Published:** 2016-02-25

**Authors:** Vanessa C. Korb, Anil A. Chuturgoon, Devapregasan Moodley

**Affiliations:** Discipline of Medical Biochemistry and Chemical Pathology, University of KwaZulu-Natal, Howard College Campus, King George V Avenue, 4001 Durban, South Africa; vanessakorb@gmail.com (V.C.K.); Devan_Moodley@hms.harvard.edu (D.M.)

**Keywords:** tuberculosis, mycobacterium, granuloma, foamy macrophage

## Abstract

*Mycobacterium tuberculosis* (MTB) is one of the most successful pathogens in human history and remains a global health challenge. MTB has evolved a plethora of strategies to evade the immune response sufficiently to survive within the macrophage in a bacterial-immunological equilibrium, yet causes sufficient immunopathology to facilitate its transmission. This review highlights MTB as the driver of disease pathogenesis and presents evidence of the mechanisms by which MTB manipulates the protective immune response into a pathological productive infection.

## 1. Introduction

*Mycobacterium tuberculosis* (MTB), the causative pathogen of the infectious and contagious disease tuberculosis (TB), is the second leading killer globally from a single infectious agent. Skin test conversion indicates that approximately one third of the global population is infected with MTB with an estimated nine million new cases and 1.5 million TB deaths in 2013 [1]. Due to the current lack of an effective vaccine, prolonged treatment periods and extensive side effects of toxic chemotherapeutic agents, research into understanding the host and bacterial role in the immune response is crucial. This is particularly true in light of drug-resistant MTB strains recognized as a potentially disastrous threat to global health.

Tuberculosis is classified as a granulomatous inflammatory condition where effector cells accumulate at the site of mycobacterial infection to form the characteristic tubercle. This physically contains the infection, suppressing bacterial replication and preventing dissemination, resulting in subclinical disease. However, the granuloma also shields the bacteria from the immune system, providing a niche of bacterial survival. Latency is considered the hallmark of protective immunity, classically attributed to the coordinated activity of the host cluster differentiation (CD)4 T-cell responses and successful in approximately 90% of infected individuals [2].

Primary active disease or reactivation is generally considered a failure of CD4 T-cell immunity. However, rather than the absence of immune activity to explain the re-emergence of a pathogen, active TB infection displays areas of intense immune infiltrate [3]. A contrasting school of thought ascribes active disease progression to MTB-mediated dysregulation of the immune response into a pathological productive infection. This review aims to highlight the role played by MTB in manipulating the host’s protective immune response during establishment, maintenance and necrotic liquidation of the granuloma facilitating transmission.

## 2. Disease Pathogenesis

MTB infection follows a well-described set of events. Briefly, bacilli are inhaled as droplets from the atmosphere with the infectious dose estimated at a single bacterium. The innate immune response is initiated when MTB in the alveolar space are recognized by pattern recognition receptors (PRRs), predominantly Toll-like receptors (TLRs) on alveolar and interstitial macrophages, as well as local dendritic cells (DCs), and, subsequently, are engulfed [4]. These antigen-presenting cells (APCs) present antigen in association with major histocompatibility complex (MHC) class II molecules to CD4 T-helper (Th) lymphocytes, and to a lesser extent, in association with MHC-I and CD1 to CD8 T-cells.

Up to the initiation of acquired immunity, macrophages remain relatively permissive to intracellular MTB. This is identified as a period of exponential bacterial replication [5]. Primed T-cells then recognize and activate macrophages to institute anti-mycobacterial killing mechanisms through the secretion of interferon (IFN)-γ and tumor necrosis factor (TNF)-α, bringing bacterial replication under control, but not eradication. These pro-inflammatory cascades culminate in the remodelling of the infection site into the granuloma. The result is a chronic infection associated with slow or non-replicating bacteria and potentially-progressive pathology [6].

### 2.1. Macrophages

Macrophages play a dual role in MTB infection. MTB targets and replicates within modified phagosomes of macrophages, employing a multitude of strategies to evade clearance by both the innate and adaptive immune systems, yet macrophages are the predominant cell responsible for MTB killing.

Infected macrophages initially respond with a vigorous pro-inflammatory and anti-microbial response [7] mediated by TLR signaling, TLR-2 being one of the major PRRs for detecting MTB [8,9]. MTB has a diverse range of TLR-2 ligands, including MTB heat shock protein (Hsp) 65, MTB Hsp70, several types of lipoproteins (LpqH, LprA, LprG, PhoS1), glycolipids (lipoarabinomannan (LAM), lipomannan (LM), phosphatidyl-myo-inositol mannosides (PIMs)) and trehalose dimycolate (TDM) [10,11,12].

Acute TLR-2 ligation enhances both innate and adaptive immune functioning, permitting containment of the infection. However, prolonged MTB-induced TLR-2 signaling has been shown to downregulate immune responses through recruitment of Foxp3 T-regulatory cells to the site of infection [13], increased interleukin (IL)-10 production [14], reduced macrophage IFN-γ sensitivity [15], inhibition of MHC class II expression [16], together with antigen processing and presentation evading recognition by T-cells [10,12]. This may be particularly important during the chronic phase of infection when T-cell immune surveillance is focused on macrophages: MTB turns a liability, namely its cell wall abundant with PRR ligands, into a mechanism for avoiding recognition by T-cells and inhibition of effector T-cell responses, facilitating the persistence of bacilli [12].

Following MTB engulfment, alveolar macrophages enter the lung interstitium establishing a site of infection [17,18]. This consequently leads to a localized pro-inflammatory cascade with production of TNF-α, IL-1, -6 and -12, together with inflammatory chemokines (CCL2, CXCL10) by the infected macrophage. The chemokine gradient recruits waves of neutrophils, natural killer cells, CD4, CD8 and γδ T- and B-lymphocytes, each producing their own complement of chemokines and cytokines, which amplify cellular recruitment and remodelling of the infection site into the granuloma [17,18,19,20,21]. TNF-α acts in a positive feedback manner to accentuate macrophage chemokine production, thereby emphasizing immune cell accumulation and granuloma formation. Persistent TNF-α production is required to sustain the chemokine gradient and, therefore, maintain the structure of the granuloma [19].

### 2.2. Dendritic Cells

DCs play a central role in the switch from an innate immune response to acquired specific immunity. MTB is phagocytosed by DCs surveying the airways, resulting in DC production of IL-12, upregulation of MHC classes I and II and CD1-associated antigens, as well as co-stimulatory molecules CD40/54/80/86. In conjunction is the acquisition of a motile phenotype resulting in migration to the proximal lymph nodes, where they prime naive T-cells [22]. Antigen-specific T-cell responses are noted in the mediastinal lymph nodes approximately 10 days following aerogenic murine infection at the earliest [23]. This is the rate-limiting step of T-cell priming.

Recent evidence shows that MTB infection of DCs inhibits their migration through the CCL19-CCR7 gradient [24]. This lag permits exponential bacterial replication, producing a high pulmonary bacterial load at the onslaught of T-cell immunity [25]. Furthermore, several MTB factors have been shown to downregulate DC function: cell envelope-associated serine hydrolase, Hip1, was shown to impair the generation of reactive oxygen species, Th1, inducing cytokine IL-12 secretion, as well as other pro-inflammatory cytokines (IL-1β, -6, -18, -23, TNF-α) via MyD88- and TLR-2/9-dependent pathways, as well as reduced CD40/CD86 expression, impairing DC maturation and antigen presentation [26]. MTB culture filtrate protein (CFP)-10 inhibits DC IFN-γ, increases IL-10 production and, consequently, suppressor pathways following the DC-T-cell interaction [27]. MTB mannose lipoarabinomannan (ManLam) has been shown to ligate DC-specific intracellular adhesion molecule-3 (ICAM 3), grabbing non-integrin dendritic cell-specific intercellular adhesion molecule-3-grabbing non-integrin (DC-SIGN), resulting in an increased expression and recruitment of suppressor of cytokine signaling 1 (SOCS-1), as well as a block of NF-kβ signaling, inhibiting IL-12 secretion and, therefore, T-cell priming [28].

### 2.3. CD4 T-Lymphocytes

CD4 T-cells are critically required for the control of primary infection and the ongoing immune surveillance of the reservoir of persistent bacilli within the granuloma from which reactivation originates [12]. This is illustrated by the fact that the selective depletion of CD4 T-cells by the human immunodeficiency virus (HIV) significantly increases TB reactivation rates to 5%–10% per life year [29]. Classically, the innate immune response to MTB is superseded by an antigen-specific Th-1 IFN-γ response. IFN-γ is a critical mediator of macrophage activation and bactericidal mechanisms. Characteristically, the induction of inducible nitric oxide synthase (iNOS) and downstream production of reactive nitrogen intermediates, together with reactive oxygen species, are toxic to MTB [30,31].

## 3. The Granuloma

The granuloma serves to physically contain the bacilli, preventing dissemination, and provides a microenvironment of optimum and localized immune communication. This leads to inhibition of the growth of the bacilli, partly by activating macrophage bactericidal mechanisms and creating an oxygen- and nutrient-deprived environment [21].

According to the central dogma, the initial granuloma consists of a core of infected macrophages surrounded by additional macrophages displaying a distinct morphology, including multi-nucleated giant cells, epithelioid cells, foamy macrophages enriched with lipid droplets [32,33], granulocytes and other mononuclear phagocytes [3,5,18,19]. In the early stages of granuloma development, the nodule undergoes marked neovascularization due to a potent pro-angiogenic vascular epithelial growth factor response. The blood vessels denote extensive lymphocyte cuffing, indicating a recruitment of lymphocytes, DCs and macrophages to the site of infection [34]. As the structure matures, it develops a significant fibrous sheath of collagen and other extracellular matrix components around the macrophage-rich center. The lymphocytic infiltrate is excluded and aggregates around the fibrous cuff, defining the periphery of the nodule [3,5,18,19].

The granuloma has several morphologically distinct forms: solid (composed of dense aggregates of infected and uninfected macrophages and lymphocytes without central necrosis), neutrophilic (extensive granulocytic infiltrates and/or central core of suppuration) and caseous (enlarged necrosis and liquefaction of dead cells in the core of the granuloma, which can progress to cavities, surrounded by a cuff of macrophages and lymphocytes) with or without fibrosis or calcification [5,19].

This solid granuloma characterizes the containment or clinically-silent stage of infection, a period of stalemate when bacillary load remains constant and the infection enters latency [5,19]. As the granuloma progresses from solid non-progressive to active cavitary lesions, there is a decrease in the number of blood vessels penetrating the nodule, facilitating caseation in the hypoxic environment [19,35]. In addition, the killing of MTB within macrophages is suggested to be severely limited, as superoxide and nitric oxide production by macrophages is inhibited by hypoxia, resulting in uncontrolled bacterial replication, anticipating granuloma break down and dissemination [36].

Retrospective histological studies of patients with active disease show marked heterogeneity in granulomas seen in a single host in different stages of progression. This indicates differential immunological processes occurring separately at each site [19,37], suggesting that TB progression from latent to active infection is a dynamic evolution determined locally at the site of the granuloma [5].

While not unique to MTB, it frequently drives the development of the granuloma. This leads to physical separation of itself within the infected macrophages at the center from the activated lymphocytes on the outskirts of the fibrous capsule. The isolated central core often serves as a site of bacterial expansion [38,39,40]. Formation of an organized granuloma is a typical host response to persistent antigens, such as mycobacterial proteins and lipids.

MTB proteins and lipids have an established granulomatous effect. MTB 6-kDa early secretory antigenic target (ESAT-6) [38] and PIM2 [41] induce matrix metalloproteinase (MMP)-9 expression in pulmonary epithelial cells neighboring infected macrophages, promoting recruitment of macrophages [42]. LM, LAM, TDM and PIMs are known to induce chemokine and pro-inflammatory cytokine production from mononuclear cells through pattern recognition receptors, such as TLR-2 [40,43,44,45,46]. These granulomatous effects provide an accumulation of uninfected host macrophages with suboptimal activation, allowing for continued mycobacterial persistence in the core while physically separated from the bactericidal activity of lymphocytes [39,40].

## 4. Immune Evasion Strategies

MTB survives and replicates within macrophages, establishing a chronic persistent infection. This is achieved by arresting phagolysosome biogenesis, thereby restricting the unfavorable intracellular environment, preventing host effector mechanisms and shielding MTB from antigen processing pathways.

A feature of phagosome maturation is acidification (pH 5.0 and lower) of the phagosome lumen. The acidic environment inhibits bacterial activity, optimizes the activity of hydrolytic proteases and ensures correct vesicular trafficking and phagosome-lysosome fusion events and degradation of pathogen into components for antigen processing and presentation, with the end result the activation of the cell-mediated immune response. Acidification is achieved by the recruitment of V-ATPases to the phagosome, which actively pumps hydrogen ions across the phagosome membrane. MTB-containing phagosomes acidify to a minimum of pH 6.4 and subsequently fail to fuse with lysosomes [47,48,49]. MTB has been shown to exclude V-ATPases from the phagosome membrane, preventing acidification [50]. This is mediated by an MTB protein tyrosine phosphatase PtpA, which binds to subunit H of the macrophage V-ATPase, inhibiting the trafficking of vesicles containing the V-ATPase complex to the phagosome [51].

The arrest of phagosome-lysosome fusion has been shown to occur due to a failure in early endosomal Rab5 to late endosomal Rab7 conversion, preventing completion of endosomal sorting and membrane trafficking. Rab conversion occurs through a calcium/calmodulin/calmodulin kinase II-dependent phosphatidylinositol-3-kinase (PI3K)/phosphatidylinositol-3-phosphate (PI3P) pathway. This cascade mediates the recruitment of the Rab5 effector early endosomal antigen 1 (EEA1) to the phagosome, triggering the fusion of phagosomes with late endosomes. Several bacterial products are observed to be involved; LAM has been shown to interfere in the calcium fluxes required for this pathway, consequently inhibiting it [52]. ManLam has been shown to block the calcium/calmodulin recruitment of PI3K to the phagosome, preventing PIP3 generation and EEA1 acquisition [48,53,54]. A bacterial PI3P phosphatase, SapM, degrades any PI3P, which evades the ManLam block [48,55]. The combined effect is inhibition of phagolysosome biogenesis.

Macrophage lysosomes are the convergent step between phagocytic and biosynthetic pathways, where antigens are processed and complexed with MHC molecules for presentation to T-cells [56]. Accumulation of MTB components in acidic late endosomal compartments optimizes processing. The MTB phagosome has limited acidification and hydrolytic activity and, therefore, a suboptimal capacity to process antigens [57]. Although the contribution of macrophage antigen presentation to the development of the adaptive immune response in TB is limited, the inhibition of phagolysosome biogenesis not only avoids MTB destruction by hydrolytic proteases, but also presentation and recognition by primed T-cells [58].

## 5. *Mycobacterium tuberculosis* Membrane Vesicles

Components of the bacterial cell wall with biological activity are shed into membrane vesicles within the MTB-containing phagosome. These vesicles are then actively trafficked out of the phagosome within the endocytic network and accumulate in multilamellar bodies in mixed micellar structures. Subsequently, the vesicles consolidate in dense lysosomal vacuoles, known as the MHC class II-enriched compartment [59]. These vesicles are released from the infected macrophage by a constitutive calcium-dependent lysosomal exocytosis into the extracellular environment. The exosomes are than engulfed by neighboring bystander cells [19,58,59,60,61]. These include uninfected APCs, such as DCs, and subsequent T-cell priming [62]. However, this also infers the extension of MTB’s reach beyond the infected macrophage and influencing the infection environment [19,61].

The predominant virulence factors found in the membrane vesicles are associated cell wall proteins of the Ag85 complex [60,63] and fibronectin attachment protein [60], phospholipids (mono-phosphatidylglycerol, diphosphatidylglycerol, phosphatidylethanolamine), glycolipids (phosphatidylinositol, PIMs, LAM, LM, TDM, phenolic glycolipid mycoside B) and lipoproteins (LprG, LprA and 19kDa lipoprotein LpqH) [40,59], each of which has immunomodulatory activity. MTB membrane vesicles are shown to contain MTB TLR-2 agonists [9,10,16,45] and stimulate the production of a range of cytokines and chemokines (IL-1β, IL-6, IL-10, IL-12, TNF-α, CXCL1, CCL3) and molecules involved in the granulomatous response (cyclo-oxygenase 2 (COX-2), MMP-9), as well as neutrophil, monocyte and macrophage recruitment [64].

## 6. Chronic Infection and Transmission

Preceding research dictates that active infection advances from failure to contain infection within the granuloma due to the impairment of the host immune response, specifically T-cell responses. HIV co-infection is the strongest known risk factor for immediate (primary) and delayed (post primary) progression from infection to active disease [65]. By this model, excessive bacterial replication results in progressive necrosis followed by caseation, liquidation of the granuloma and cavitation into the bronchi, releasing bacilli. This necessitates the development of a productive cough, resulting in aerosol transmission of infectious MTB [2,19].

However, contemporary histological analysis of biopsies from patients with untreated TB demonstrated that reactivation originated from areas of lipoid pneumonia. Within these areas, there was sequestration of abundant lipid-rich macrophages, termed foamy macrophages (FM), accompanied by bronchial obstruction. Furthermore, infection was restricted to the FM, and bacilli were predominantly found within the lipid droplets. Cavities originated from tuberculosis pneumonia in individuals who had no histological evidence of caseating granulomas [33,66,67]. Whereas previously tuberculosis pneumonia was regarded as a consequence of cavitation, this suggests that TB progression is a pneumonic process, rather than a granulomatous one [66].

### 6.1. The Foamy Macrophage

MTB evades the immune response sufficiently to allow persistent infection, but simultaneously promotes sufficient immunopathology to ensure its transmission. A more recent school of thought suggests that MTB dysregulation of host lipid synthesis and lipid accumulation is pivotal in transition from latent to active infection. The foamy macrophage, often observed in pathologies with chronic pro-inflammatory stimulus, is pin pointed as the key player in sustaining persistent bacterial infection and the primary driver of pathology, leading to cavitation and transmission [5]. Membrane vesicles inoculated into a murine model produced a granulomatous response, including the generation of FMs [68].

Macrophages are converted into foam cells by an imbalance in the influx and efflux of low density lipoproteins (LDL) [69]. LDL particles contain phospholipids, triacylglycerides (TAGs) and cholesterol. Following the breakdown of the LDL within the macrophage, the majority of the phospholipids and TAGs are metabolized. However, the cholesterol is transported into the cytosol and either esterified and sequestered into lipid bodies formed within the endoplasmic reticulum or it is pumped out of the cell by ATP-binding cassette transporters [5]. The evolution from macrophage to foamy macrophage is through the accumulation and progressive transformation of lipid bodies [32].

The FM is in its self a pro-inflammatory cell [70]. Lipid bodies have been shown to play a significant role in the inflammatory response of granulomas [71]. In a series of human biopsy samples, it was shown that these FMs were systematically located at the interface between the histiocytes and the necrotic core in each biopsy. Moreover, FMs were only observed in necrotic lesions, and their presence correlated to the onset of necrosis [5,33,66,67]. The spatial and temporal relationship between FMs and the necrotic center of the granuloma indicates a causal association. Lipids from the caseating debris in human TB granulomas are identified to predominantly include cholesterol, cholesteryl ester and TAGs; reflecting a potential sequestration from lipid droplets in FMs [5]. In addition, MTB-induced FMs displayed an elevated continual secretion of TNF-α, a strong inducer of necrosis, at both the protein and molecular levels. However, it has been cautioned that this spatial location may also suggest that FMs may be associated with necrosis as a consequence of clearing the necrotic debris, including lipoproteins, by phagocytosis [33].

In a human granuloma model, FMs lose their ability to mediate phagocytosis accompanied by reduced anti-mycobacterial mechanisms [33]. Furthermore, murine FMs are demonstrated to have reduced antigen processing capacity [72], as well as suppress effector T-cells during *in vivo* MTB infection [73]. FMs were also found to secrete high levels of TGF-β, which can cause apoptosis of immune effector cells [74], as well as the production of high levels of iNOS, which suppresses T-cells in murine TB infection [75]. Together, these findings indicate a role of FMs in immune modulation and sheltering of MTB.

FMs can be generated by the phagocytosis of dying cells (suggesting their presence on the outskirts of the necrotic core). More specifically, the membranes of the dying cells contain large amounts of arachidonic acid (AA) precursors and associated enzymes. In inflammatory leukocytes, AA, a precursor of inflammatory mediators, is stored in lipid bodies in an esterified form. AA-mediated metabolic pathways have been implicated in the inflammatory response to MTB by either promoting or suppressing inflammation through the production of prostaglandins (PG), lipoxins and leukotrienes [71,72,76].

In a Bacillus Calmette–Guerin (BCG)-infected murine model, an increase in macrophage lipid bodies was correlated to increased generation of prostaglandin PGE2 and localization of the eicosanoid forming enzyme COX-2 within lipid bodies. PGE2 is a potent suppressor of Th1 responses, as well as TNF and nitric oxide production and, therefore, macrophage response, favoring MTB persistence. Moreover, the lipid bodies were identified as the predominant site of PGE2 synthesis within activated macrophages. The authors concluded that these lipid bodies serve as signaling platforms with a direct impact on the capacity of the host cell to produce eicosanoids and, in this way, contribute to the modulation of disease pathogenesis [71].

Recently, it has been demonstrated that FM generation is specifically induced by oxygenated mycolic acids (oxygenated ketomycolic) and hydroxyl mycolic acids, of virulent mycobacterial strains. This was independent of the appearance or stage of disease, but dependent on direct contact of the macrophage with the bacterial components [33]. This effect was demonstrated by both whole bacilli and isolated lipids, indicating that oxygenated mycolic acids can either be expressed at the cell wall or released into membrane vesicles in order to perpetrate their biological activity in both infected and uninfected macrophages [33]. In support of this, BCG was shown to induce FM generation in a TLR-2-dependent manner of both infected and uninfected bystander cells, indicating that bacilli ingestion is not a pre-requisite of FM generation [71].

Mycolic acids form a significant and characteristic component of the MTB, constituting 40%–60% of its dry weight [77]. The most widely-characterized mycolic acid containing MTB compound is TDM. Until recently, mycolic acids were only considered indirectly virulent as part of the MTB cell envelope, yet now are regarded as virulence factors facilitating persistence in chronic infection through the accumulation of host lipids and FM generation at the site of infection [33].

In macrophages derived from the human monocytic THP-1 cell line, lipid body accumulation was induced by MTB through modulation of host lipolysis of neutral lipids. This was achieved by diverting the glycolytic pathway towards ketone body synthesis, specifically 3HB, allowing feedback activation of the host cell-specific anti-lipolytic G-protein-coupled receptor GPR109A. Dysregulated lipid homeostasis caused a decrease in intracellular cyclic adenosine monophosphate (cAMP) and inhibition of cAMP-dependent signaling pathway and subsequent lipase action, permitting the generation of lipid bodies. This feedback loop was specific to virulent strains of mycobacteria and was observed to be enforced by MTB ESAT-6. Furthermore, accumulation of lipid bodies correlated with the inhibition of autophagy and lysosome acidification [78].

### 6.2. MTB-Host Lipid Metabolism

Intracytoplasmic lipid inclusions have been identified in bacilli isolated from positive sputum of TB patients [79]. Based on *in vitro* studies, MTB-containing phagosomes migrate towards the lipid droplets and undergo mycobacterium-driven fusion; lipid bodies are released into the phagosome combined with a thinning of the bacterial cell wall [33,80]. The major component of lipid inclusions was identified as host TAGs, providing direct evidence that host TAGs are used for lipid metabolism within MTB [79,81]. The hypervirulent MTB Beijing strains have been linked to the accumulation of TAGs *in vitro*, substantiating the significant role of lipids in the MTB life cycle [82]. It has been observed that MTB in FMs, which accumulate lipid inclusions, lose acid-fast staining and become phenotypically-resistant to the two frontline TB drugs, Rifampicin and Isoniazid, with upregulation of MTB genes involved in dormancy and lipid metabolism; characteristic of latent bacilli and indicative that FMs support MTB in a dormant state [33,80,81].

Once within MTB, it is suggested that lipid body TAGs are hydrolysed to fatty acids mediated by MTB lipases present on the cell wall, such as LipY [80,81]. These fatty acids were shown to be imported into the MTB cytoplasm, where they were resynthesized into bacterial TAGs by triacylglycerol synthase 1 (TGS1) [81,83]. A transcriptional link has been identified between the dormancy regulator gene, *DosR*, and *tgs*1 [84].

In several infectious diseases, FMs have been associated with the nutritional requirements of the pathogen. Lipids have been shown to be a major source of energy for latent bacilli [85] and a source of building materials for the cell wall during reactivation and replication [32,86]. MTB reactivation from hypoxia-induced dormancy is observed to involve TAG hydrolysis by MTB lipases. Bacterial TAGs accumulated during dormancy, through the action of TGS1, were rapidly depleted upon re-aeration and reactivation [87]. This implies that MTB can manipulate host cell metabolism, inducing the generation of FMs and intracellular lipid droplets to satisfy the nutritional and structural requirements of the bacterium.

In chronically-infected lung tissue, fatty acids are indicated as a source of carbon and energy for MTB metabolism [33,88]. MTB found in FMs in the peripheral lymphocyte cuff of the granuloma were positive for expression of isocitrate lyase 1 (ICL1) [89]. This enzyme is the gating enzyme to the glyoxylate shunt, essential for fatty acid metabolism and activated when organisms survive on fatty acids as their limiting carbon source. Disruption of the *icl1* gene attenuated bacterial persistence and virulence in the chronic stage of infection in IFN-γ activated macrophages *in vitro* [85].

In addition, MTB’s capacity to metabolize cholesterol for carbon and energy has been observed. The utilization of cholesterol, represented by the MTB mce4 cholesterol import system, is essential for maintaining persistence in the lungs of chronically-infected mice, as well as in IFN-γ-activated macrophages. This observation in activated, but not resting, macrophages was suggested to be due to the abundance of alternate carbon sources in resting macrophages where the bacteria is localized in the endocytic network. IFN-γ macrophage activation causes maturation of the MTB phagosome and its restriction from recycling endosomes, therefore limiting the available carbon. Nutrient restriction is a specific IFN-γ-mediated defense mechanism, and MTB utilization of TAGs and cholesterol is a way to circumvent the host response [6]. A murine model defective in *mce4* expressed a chronic persistence defect similar to that seen in the *icl1* defect [6,85]. MTB-mediated cholesterol metabolism increases the propionate pool within the bacterium [90,91]. Carbon intermediates, such as propionyl-CoA, can be used as building blocks for the bacterial cell wall upon reactivation and replication. The cell wall components act as structural, as well as virulence factors involved in modulation of the host immune response [40,59].

Taken together, the ability of MTB to sustain a chronic infection is critically linked to its ability to acquire and utilize host lipids [6].

### 6.3. Hypoxia and Lipid Metabolism

Chromatin immunoprecipitation and subsequent sequencing, together with system-wide profiling during normoxia, hypoxia and re-aeration, have uncovered interconnections between the hypoxic response, lipid catabolism, lipid anabolism and the production of cell wall lipids. More specifically, transcriptional responses linking hypoxic adaption, lipid and cholesterol degradation and lipid biosynthesis, such as the linking of *tgs1* and *DosR*, were identified. Also noted was an increase in free mycolic acids, responsible for FM generation, at the onset of hypoxia and a decrease on re-aeration. Changes in oxygen status result in alteration in the expression of almost one third of MTB’s genes [84].

The relationship between MTB and FMs provides a rich source of nutrients to the bacteria during chronic infection, while shielding it from bactericidal and inflammatory pathways [33,71]. In the core of the granuloma, hypoxia induces a non-replicative state characterized by phenotypic resistance to anti-TB medication, as well as detection by acid-fast staining, with a shift to lipids, such as TAGs and cholesterol, as a major nutrient source [81,84]. The accumulation and catabolism of host lipids is linked to the biosynthesis of bacterial lipids for energy, cell wall components and virulence factors, part of the well-documented metabolic reprogramming of the host cell [84,91].

Using positron emission tomography–computed (PET-CT) scanning, it has been shown that areas of consolidation and areas immediately surrounding cavities within the lungs of pulmonary TB patients show marked hypoxia. This was associated with an MTB-induced 3.5-fold increase in MMP-1 expression and 2.1-fold increase in MMP-9 expression compared to normoxic cells. In addition to cellular recruitment, MMPs have been implicated in the breakdown of the extracellular matrix during transmission. MTB has been shown to upregulate MMP-1 and -9 expression, driving proteolytic cleavage of the extracellular matrix [92,93]. Further, in MTB-infected primary human-monocyte-derived macrophages and respiratory epithelial cells, hypoxia caused a 250-fold and 95-fold increase in MMP-1 gene expression, respectively. This occurred in a NF-κB and hypoxia inducible factor-1α-dependent manner, indicating that hypoxia promoted extra-cellular matrix destruction [94]. Together, these findings highlight the potential role played by hypoxia in initiating latent bacterial persistence at the transcriptional level and facilitating events leading to cavitation.

### 6.4. Host Cell Death

Virulent MTB survive within macrophages by preventing apoptosis, phagosome maturation and antigen processing, creating a niche where bacteria remain metabolically active and capable of replication [95]. However, MTB would benefit from cell death once a high intracellular bacterial load has been reached, allowing for dissemination of the bacilli. Necrosis is the preferred MTB exit strategy, promoting inflammation and disease progression [96,97].

MTB has been shown to cause plasma membrane microdisruptions in the infected macrophage. Repair of these lesions by exocytosis of endomembranes is required for preventing necrosis and promoting apoptosis. This is achieved by the fusion of Golgi and lysosome-derived vesicles with the plasma membrane, the process being dependent on PGE2 [98]. In the late stages of infection, macrophages infected with virulent MTB strains preferentially synthesize lipoxin A4 (LXA4), which inhibits apoptosis and promotes necrosis, shielding the MTB from the innate immune system. The production of LXA4, which blocks PGE2 biosynthesis by downregulation of *COX*-2, is a natural anti-inflammatory immune mechanism. This is exploited by pathogenic MTB at the infection site, inhibiting plasma membrane repair, driving inflammation and necrotic cell death [98,99]. Necrotic death of FMs within the granuloma leads to the accumulation of lipid debris at the core of the liquefying granuloma forming the caseum [5].

In this way, MTB-mediated dysregulation of host cell lipid metabolism drives the tissue response, resulting in necrosis and caseation. Moreover, MTB subversion of the macrophage response brings about late stage damage in the chronic phase of infection required for transmission and completion of the MTB life cycle [5].

## 7. Concluding Remarks

Despite centuries of research, the relationship between MTB and the host immune response remains enigmatic. Current investigations are beginning to elucidate the mechanisms employed by MTB in the manipulation of protective immunity from infection to transmission. A new perspective identifying MTB as the administrator of disease pathogenesis engenders novel therapeutic opportunities and the need to reassess experimental design and research goals.

## Abbreviations

AA, arachidonic acid; APC, antigen-presenting cell; BCG, Bacillus Calmette–Guerin; cAMP, cyclic adenosine monophosphate; CD, cluster differentiation; COX-2, cyclo-oxygenase 2; DC, dendritic cell; EEA1, early endosomal antigen 1; ESAT-6, 6-kDa early secretory antigenic target; HIV, human immunodeficiency virus; ICL1, isocitrate lyase; iNOS, inducible nitric oxide synthase; IL, interleukin; IFN, interferon; LAM, lipoarabinomannan; LDL, low density lipoprotein; LM, lipomannan; LXA4, lipoxin A4; ManLam, mannose lipoarabinomannan; MHC, major histocompatibility complex; MMP, matrix metalloproteinase; MTB, *Mycobacterium tuberculosis*; PG, prostaglandin ; PI3K, phosphatidylinositol-3-kinase; PI3P, phosphatidylinositol-3-phosphate; PIM, phosphatidyl-myo-inositol mannosides; TAG, triacylglyceride; TB, tuberculosis; TDM, trehalose dimycolate; TGS1, triacylglycerol synthase 1; Th, T-helper; TLR, Toll-like receptor; TNF, tumor necrosis factor; ICAM, Intercellular adhesion molecule; SOCS-1, suppressor of cytokine signaling 1; PET-CT, positron emission tomography–computed.
